# Performance of patients with frontotemporal lobar degeneration on artistic tasks: A pilot study

**DOI:** 10.1590/S1980-57642014DN81000011

**Published:** 2014

**Authors:** Maria Cristina Anauate, Valéria Santoro Bahia, Ricardo Nitrini, Marcia Radanovic

**Affiliations:** 1 Behavioral and Cognitive Neurology Unit, Department of Neurology, University of São Paulo School of Medicine, São Paulo, Brazil.; 2 Cognitive Disorders Reference Center (CEREDIC) - Hospital das Clínicas - University of São Paulo School of Medicine, São Paulo, Brazil.

**Keywords:** art therapy, executive function, visual perception, frontotemporal lobar degeneration

## Abstract

Several studies have addressed visuospatial and executive skills in artistic activities in Frontotemporal Lobar Degeneration (FTLD) and Alzheimer's disease (AD).

**Objective:**

To investigate the performance of FTLD patients compared to controls on two artistic tasks.

**Methods:**

Four FTLD patients with mean age of 57 (8.7) years and schooling of 12.2 (4.5) years plus 10 controls with mean age of 62.9 (8.6) years and schooling of 12.3 (4.6) years, were assessed using the Lowenstein Occupational Therapy Cognitive Assessment (LOTCA) and by a three-stage artistic protocol including visual observation, copying and collage, based on a Sisley painting.

**Results:**

FTLD patients had lower scores than controls on Visuospatial Perception, Copy, Collage, Examiner's Observation, and Total, showing distinct patterns of performance according to FTLD sub-type: semantic PPA, nonfluent PPA and bvFTD.

**Conclusion:**

FTLD patients presented impairment in the visuospatial and executive skills required to perform artistic tasks. We demonstrated that the application of the instrument as a complimentary method for assessing cognitive skills in this group of patients is possible. Further studies addressing larger and more homogeneous samples of FTLD patients as well as other dementias are warranted.

## Abstract (pt)

Vários estudos relatam as habilidades visuoespaciais em atividades artísticas em Degeneração Lobar Frontotemporal (DLFT) e Demência de Alzheimer (DA).

**Objetivo:**

Investigar as alterações no desempenho de pacientes com DLFT comparados a controles em tarefas artísticas.

**Métodos:**

Quatro pacientes com DLFT (idade média de 57 (8,7) e escolaridade de 12,2 (4,5) anos) e 10 controles (idade média de 62,9 (86) e escolaridade de 12,3 (4,6) anos) foram avaliados através da Bateria de Avaliação Cognitiva de Terapia Ocupacional Lowenstein (LOTCA) e um protocolo de arte em três estágios: observação visual, cópia colorida e colagem baseada em uma pintura de Sisley.

**Resultados:**

Os pacientes com DLFT tiveram menores pontuações em Percepção Visoespacial, Cópia, Colagem, Observação do examinador e Total, mostrando distintos tipos de desempenho nos subtipos de DFT variante comportamental, APP não fluente e APP semântica.

**Conclusão:**

Os pacientes com DLFT apresentaram prejuízo nas habilidades executivas e visuoespaciais necessárias ao desempenho nas tarefas artísticas propostas. Evidenciamos ser possível a aplicação deste instrumento como método de avaliação complementar de habilidades cognitivas neste grupo de pacientes. Novos estudos em um grupo maior e mais homogêneo de pacientes com DLFT, bem como em outras demências, são necessários.

## INTRODUCTION

Artistic production involves the visual system, praxis, memory and executive functions, as well as emotional processes, creativity and inspirations that give rise to art.^1^ In recent years, studies in the fields of Neurology, Psychology, Psychiatry and Cognitive Sciences have enabled different and complementary perspectives of art production and related processes in the brain.

Research on visuospatial perception, visual imagery, motor memory and artistic processes in the brain have been performed in different neurodegenerative diseases. Studies involving patients with dementia have contributed to our understanding of brain regions implicated in art-making, but the current body of knowledge in this area remains small.^2,3^ Research on art and the brain is still scarce in either retrospective or prospective case-controlled studies employing quantitative methods with groups of patients.^2^

Frontotemporal lobar degeneration (FTLD) is a complex of predominantly cortical degenerative diseases, usually pre-senile, that is characterized by deterioration in personality and cognition associated with frontal and temporal lobar atrophy. In this type of dementia, behavioral, executive, and language alterations predominate over the episodic memory and visuospatial impairment as found in AD.^4,5^

Some studies have addressed visuospatial skills in artistic activities at early to moderate FTLD stages. There are case reports of artists with Alzheimer's disease (AD) whose performances underwent changes during the disease course. Few studies have addressed art production in stroke patients.^3,6-10^ Rankin et al.^2^ published the first study with a quantitative method for evaluating art production in FTLD patients, comparing their performance to that of AD patients and normal elderly subjects.

Several studies have documented alterations in artistic expression in clinical subtypes of FTLD: behavioral FTD (bvFTD) and Primary Progressive Aphasia (PPA). Miller et al.^11^ studied the emergence of artistic abilities in FTLD. Maintained creativity in FTLD has been observed in visual art and music, but not in writing and poetry.^12,13^ Despite the existence of individual patients displaying increased artistic production, apathy leading to diminished creativity is the most typical finding in FTLD patients.^14^

The aim of this study was to compare the artistic and visuospatial performances in patients with FTLD (semantic PPA, nonfluent PPA and bvFTD) versus normal subjects.

## METHODS

**Participants.** The study group comprised fourteen subjects: four FTLD patients (two bvFTD, one semantic PPA and one non-fluent PPA) and ten controls. Patients were recruited from a Behavioral and Cognitive Neurology outpatient unit of a tertiary hospital, and controls were recruited from the community.

The inclusion criteria for FTLD were according to Neary et al.^4^ The control group was composed of elderly subjects who fulfilled the Mayo Clinic's normative criteria (MOANS) for inclusion in neuropsychological studies.^15^

The exclusion criteria for all groups were: severe visual deficits, severe constructional apraxia, hemianopia, neglect, musculoskeletal disorders and illiteracy. None of the subjects had previous experience in formal studies of art. All patients' representatives signed an informed consent prior to enrollment in the study.

**Procedures.** All subjects were evaluated using the Mini-Mental State Examination (MMSE),^16^ the Clock Drawing Test (CDT),^17^ the Pfeffer Functional Activities Questionnaire (P-FAQ),^18^ and the Geriatric Depression Scale,^19^ prior to enrollment in the study. Subjects in the control group were admitted only if they attained a score greater than or equal to the cut-off scores defined for the Brazilian population on the MMSE.^20^

Subjects were then assessed using the Lowenstein Occupational Therapy Cognitive Assessment battery (LOTCA)^21^ and by an art protocol created by the main investigator (M.C.A.) using a three-stage artistic task:

[1] visual observation;
[2] colored copy;
[3] collage using shapes cut from colored paper; all tasks were based on a reproduction of a Sisley's painting.

Patients were instructed to look at the painting and objectively describe the scene as thoroughly as possible; patients were also encouraged to describe their own feelings and memories elicited by the scene (stage 1 - visuospatial perception). Next, participants were given a sheet of paper and colored pencils, and asked to copy the scene as accurately as possible (stage 2 - colored copy). Finally, patients were supplied with colored papers, scissors, and glue, and instructed to make a collage reproducing the same picture (stage 3 - collage). The tasks were performed individually in a silent room during a single session. Time was computed, but there was no time limit for the performance of the tasks.

Each task in the art protocol was scored on a scale of 1 to 4 to generate quantitative results. The criteria for constructing the score sheet took into account aspects of the visuoconstructional skills involved in artistic production and evaluation of visual art, according to Drago et al.^22^ The sub-items of the first stage (visuospatial perception) were based on identification of figures, scenery, colors, shapes, depth and spatial location. Items in the second stage (colored copy) were based on the analysis of the quantity of design elements, proportions, colors, correct use of paper, gestalt and richness of details. The sub-items of the third stage (collage) were based on the analysis of organization and planning, ideational praxis, quantity and richness of elements represented, arrangement of cuts, colors and analytical/synthetic balance. In addition to these three stages, the examiner's observation also included aspects of attention, cooperation, interest and judgment of patients in relation to the task, which were also scored. The criteria for evaluation and scoring were based on previously published data regarding art production alterations described in subjects with neurologic impairment (Appendix 1).

The copy and collage were scored by a professional who did not participate in the subjects' evaluation.

A descriptive statistical analysis was performed for each demographical and clinical variable studied.

## RESULTS

FTLD patients had a mean age of 57±8.7 years and mean schooling of 12.2±4.5 years; two patients were male and two were female. The control group had a mean age of 62.9±8.6 years and mean schooling of 12.3 ±4.6 years; two patients in this group were male and eight were female. FTLD patients were younger than controls, and the control group was composed predominantly of women. Clinical data of the sample are displayed in Table 1; as expected, the FTLD group performed poorer on cognitive and functional tasks. Table 2 displays the performance of the groups on the LOTCA battery; FTLD patients performed poorer than controls on all subtasks of the battery, especially in orientation, visuomotor organization and thinking operations. The performance of the subjects on the art protocol is also shown in Table 2. FTLD patients performed poorer than controls on all stages of the composition.

**Table 1:** Performance of subjects on the cognitive and functional screening

Variable	FTLD (4)M (SD)	Controls (8)M (SD)
MMSE	18.8 (5.1)	29.2 (1.1)
CDT	4.2 (3.5)	9.7 (0.5)
P-FAQ	19 (2.9)	0.6 (0.8)
GDS	5.5 (5)	2.2 (1.7)

**Table 2:** Performance of subjects on the LOTCA battery and art protocol.

Cognitive assessment	FTLD (4)M (SD)	Controls (8)M (SD)
**LOTCA battery**		
Orientation (8)	5.2 (2.5)	8 (0)
Visual and spatial perception (20)	17.5 (2.3)	20 (0)
Praxis (4)	3.2 (0.5)	4 (0)
Visuomotor organization (28)	18 (3.9)	25.5 (3.5)
Thinking operations (26)	11.2 (4)	23 (3.3)
Attention/Concentration (4)	3 (1.4)	4 (0)
Total (90)	58.2 (6.6)	84.5 (6.4)
**Art protocol**		
Observation and description (20)	13.2 (3.7)	20 (0)
Copy (24)	17.5 (5.2)	22.7 (2.2)
Collage (28)	20.5 (6.4)	26.6 (2.2)
Examiner's observation (24)	20.2 (5.5)	23.5 (0.9)
Total (96)	71.5 (13.8)	92.8 (4.3)

### Case reports and qualitative evaluation

*Patient 1 (bvFTD)* - 64-year-old, female professor and scholar in languages, produced a copy with adequate arrangement of elements, complete, but showing few details; her composition was primary and simplified, with inadequate proportions. On the collage, she had great difficulty, with very few elements and very defective proportions. Her execution was rapid and impulsive, as she continuously tried to leave the room during the examination.

*Patient 2 (bvFTD)* - 44-year-old, male lathe operator, showed a fragile, scattered and impoverished copy, without gestalt. His collage was incomplete and poor with very few elements and inadequate proportions. The patient was collaborative, although lethargic and slow, with little expression and communication.

*Patient 3 (semantic PPA)* - 63-year-old, male physician, produced a copy using only lines, no painting, but his drawing was complete and proportional, although childish. On the collage, again, he performed poorly, but maintaining correct proportions. He appeared to enjoy the task, despite his apparent apathy without verbal communication.

*Patient 4 (non-fluent PPA)* - 60 year-old, female nurse, produced a copy in which the elements were well placed, rich in details, but with some open spaces, voids. On the collage, she had great difficulty in construction, with open, loose elements without gestalt, although still consistent with the model.

## DISCUSSION

Studies in the literature describe changes in artistic of FTLD expression that are manifested in the early stages, especially in patients with involvement of the left temporal lobe, impairments in language and increased right-hemisphere based visual processing.^14^

The main finding of our study was that FTLD patients showed profiles of visual art production that could be differentiated both quantitatively and qualitatively from controls. Drawings produced by FTLD patients tended to display a simpler composition, and both copy and collage were poorly and rapidly executed. FTLD subjects altered the proportion in relation to the model with use of more muted and cold colors and fewer details.

The assessment of our sample using the LOTCA battery aimed to demonstrate the abilities that were impaired in our patients, through a formal cognitive test. Although it is not possible to achieve an exact correlation between the LOTCA and our art protocol domains, we can reasonably infer that the impairments in visuospatial perception and organization, as well as thinking operations (the most affected domains in LOTCA) might well explain the poor performance of FTLD patients on tasks such as observation and description, copy and collage. The art protocol is a more ecological instrument, based on a productive task, and is in keeping with the current trends for functional evaluations.

Copy was selected as one of the tasks in our art protocol due to findings in the literature that point to the preservation or spontaneous emergence of copying abilities in FTLD^23^ that persist for a long period during the course of the disease; this task enables the observation of complex neuropsychological functions such as visuoconstructional and executive functions.

Collage is a more complex task, as it requires the individual to make a synthetic analogy of the composition; it is not literal, it does not have a baseline as a supportive path, and the borderlines are defined by clipping the paper (rather than drawing lines and contours). Collage also requires a synthesis of colors that must be recognized in a comprehensive and succinct manner. Cuts must generate chromatic geometric pieces that will be organized in superimposed planes (figure-background). Details must give way to a more global vision of the composition. The process of analysis-synthesis in this task is continuous, involving abstraction of geometric shapes grouped in a final synthesis consistent with the model, requiring much more organization, constructional planning and spatial reasoning than copying. Sustained attention is greatly required in this task, and the complexity of the model acts as a distractor factor.

Qualitative evaluation of patients' performance is also an important step of every cognitive evaluation, as it can reveal subtleties in the individual's behavior that a quantitative method is not able to afford. Hence, our art protocol proposes the sub item "Examiner's observation" that although also scored, allows for a more accurate analysis on "how" patients behave. To illustrate this, we have given a brief description of our patients' behavior and a qualitative analysis of their art works.

We observed that performance in patients with bvFTD was impaired by behavioral disorders, in particular impulsivity and intolerance regarding the duration of the tasks; executive dysfunction also hampered planning and organizing the steps of each task, an effect that was more pronounced in collage.

The "emergence" of artistic ability in FTLD has been reported in the literature.^11,13^ However, these "new acquired" abilities are better explained by a lack of inhibitory mechanisms due to damage to the prefrontal cortex leading to the liberation of involuntary movements than by actual development of creativity abilities.^24^

According to the literature, bvFTD patients are described as producing more primitive, childish drawings and paintings, with aberrant and bizarre themes, less elaborate and more disordered compositions (due to a lack of task-monitoring). They use less active mark-making. The content of artistic expression is the occurrence of mood changes, agitation and late psychosis characterizing the course of the disease.^2^ Semantic PPAs use more lines than painting, because they tend to rely on the formal elements rather than in the picture content (that they do not fully recognize) and their works appear to be "deprived of meaning".^12-14^ Non fluent PPAs are able to produce drawings and paintings of better quality. In our sample, impersistence (patient 1) and apathy (patient 2) were factors that negatively impacted on subjects' performances; patient 3 (semantic PPA) exhibited the described pattern of "line-based" production, while patient 4 (non-fluent PPA) presented the most satisfactory performance. BvFTD and semantic PPA patients used a simpler composition and altered proportions when compared to the model; they also employed muter (less bright) and colder colors and fewer details. The patient with nonfluent PPA showed more difficulty on constructional ability in the collage, especially in closure, but her production corresponded better to the model.

The use of artistic tasks in controlled experimental and descriptive studies of brain lesions may contribute to the understanding of the underlying processes involved in art production while also providing a complementary method of evaluation of neuropsychological functioning in different types of dementia. Furthermore, an increased comprehension of such processes may help to optimize rehabilitation efforts by means of art therapy in dementia.

In conclusion, this is an exploratory study of artistic abilities that may be a useful alternative tool in the assessment of cognitive dysfunction in different types of dementia. Limitations of our study are the small number of cases, which did not allow statistical analysis, and the fact that our patients had moderate to severe dementia at the time of assessment. Studying patients in early stages of dementia might provide further insight into different performance profiles and help us to understand the pattern of cognitive deterioration specific to each form of FTLD. The structured study of artistic abilities might be a useful alternative tool in the assessment of cognitive dysfunction in FTLD patients in which traditional neuropsychological testing may prove difficult due to behavioral disturbances.

We believe that further studies exploring expressive and still intact abilities in dementia patients can provide important frameworks for non-pharmacological and rehabilitation interventions. Studies with larger samples, exploring neuroimaging data and clinical interventions with a focus on neuropsychiatric disorders in FTLD and other forms of dementia are warranted.

## APPENDIX 1: ART PROTOCOL - PROCEDURES AND SCORING The sub items are scored on a scale of one (minimum) to four (maximum), except for sub item I.2 (yes or no answers). In the observation and description task, the patient answers the questions formulated by the examiner and the score is given based on the responses. In the tasks of copying and collage, the examiner asks the patient if he has finished the task and then proceeds to scoring. The examiner should be attentive throughout the course of the procedure protocol to those points described in sub item IV. The duration and number of sessions must be registered.

- **OBSERVATION AND DESCRIPTION OF THE PICTURE**
  **1. Visuospatial perception**
  - Identification of figures and scenery (a landscape of a village on the bank of a river, composed by a bridge, houses, river, trees, boat, people, and vegetation – total of 8 elements):
    1. does not identify any element
    2. identifies 1 to 2 elements and/or includes others (non-existent)
    3. identifies 3 to 5 elements
    4. identifies 6 to 8 elements
  - Identification of colors (blue, green, beige, white, brown, orange, yellow, pink and purple - 9 colors):
    1. does not identify any colors
    2. identifies 2-4 colors and/or includes non-existing colors
    3. identifies 3-5 colors
    4. identifies 7-9 colors
  - Identification of shapes: The examiner shows an alternative picture and asks the patient to point to the circles, triangles, rectangles and trapezoids – 8 elements; then the patient is asked to do the same in the protocol image. If the patient has language difficulties, a matching procedure may be employed to assure that the patient recognizes the shapes.
    1. does not identify any shape
    2. identifies only 1 shape with difficulty
    3. identifies 2-4 shapes
    4. identifies 5-8 shapes
  - Identification of depth (perspective): The examiner shows an alternative picture and asks the patient to describe what is in the foreground, center and background in the picture; then the patient is asked to do the same in the protocol image (the bridge and river are on the 1^st^ plane, the vegetation in the 2^nd^ plane, the houses in 3^rd^ plane, and the sky in the 4^th^ plane).
    1. does not identify any plane
    2. identifies only the 1^st^ plane
    3. identifies 2 to 3 planes
    4. identifies 4 planes
  - Identification of spatial location:
    1. does not identify the location of any elements
    2. identifies only those elements that are at the right side of the image
    3. identifies the locations, but does not describe all elements
    4. identifies all elements in their locations: left, right, on top and on bottom
  **2. Impact of the image on the subject**
  - These phenomena may or may not appear during the interaction of the patient with the image.
    – interpretation: Yes = 0 No = 1
    – inclusion of internal (personal) contents: Yes = 0 No = 1
    – fantasy: Yes = 0 N = 1
    – emotion: Yes = 0 N = 1
    – coldness and neutrality: Yes = 0 No= 1
- **COLORED COPY**
  - Drawing and painting:
    1. does not draw
    2. includes non-existing elements
    3. depletion of elements (draws only 2 elements)
    4. drawing and painting consistent with the model
  - Proportion of forms / planes:
    1. does not draw
    2. draw with distorted proportions
    3. draws all the elements in the same size
    4. draws with proportional sizes
  - Use of color in painting:
    1. does not use the colors of the model
    2. uses the colors of the model, but introduces non-existing colors
    3. uses fewer colors (from 2 to 4) than existing in the model
    4. use all correct colors
  - Use of the paper sheet:
    1. does not draw
    2. Uses only part of the sheet (top / bottom or left / right)
    3. Uses only the center of the sheet (or the opposite)
    4. Uses the entire sheet
  - Gestalt
    1. drawings not consistent with the model
    2. closure impairment (leaves the elements "open")
    3. closure with difficulty and hesitation
    4. closure consistent with the model
  - Richness of details
    1. absence of details, just the basic shapes of the elements
    2. inclusion of repetitive details or keeps fixed on one detail
    3. inclusion of few details
    4. adequate inclusion of details
- **COLLAGE**
  - Organization and planning:
    1. performs the task randomly
    2. tries to organize the papers, but fails and proceeds randomly
    3. organizes the papers by color, but becomes disorganized when performing the collage
    4. organizes the papers and performs the collage adequately
  - Ideational praxis
    1. unable to perform the cutting and pasting
    2. performs only the cutting or the pasting
    3. performs both cutting and pasting, but randomly
    4. performs both cutting and pasting adequately
  - Number of elements represented:
    1. does not perform the collage
    2. represents one element
    3. represents 2 to 4 elements
    4. represents 5 to 7 the elements
  - Arrangements of forms on the paper sheet
    1. arranges the paper scraps in an incoherent and random manner
    2. arranges the paper scraps grouped in a small surface
    3. overlaps elements or extends beyond the edges
    4. arranges the paper scraps adequately according to the model
  - Use of colors
    1. uses only one color
    2. uses 2-4 colors
    3. uses more than 4 colors, but not corresponding to the model
    4. uses all colors adequately
  - Richness of elements
    1. repetition and fixation in elements
    2. represents elements with poor details, but correctly
    3. represents elements without relevance to content of motif
    4. represents relevant and adequate details
  - Balance in analytic-synthetic aspects during performance
    1. does not finish the collage, gets lost in the details
    2. separates the paper scraps for all elements, but represents only a small part of the model
    3. represents the model rapidly and concisely, without attention to the details
    4. depicts the entire model with all details
- **EXAMINER’S ADDITIONAL OBSERVATIONS**
  - Attention during the procedure
    1. inattentive, requires continuous repetition of instructions and stimuli to perform the tasks
    2. maintains attention for short periods
    3. is able to sustain attention, but sometimes needs to be reoriented
    4. maintains good attention throughout the procedure
  - Collaboration
    1. uncooperative, unable to perform the procedure
    2. a little cooperative, performs only part of the task
    3. cooperative, but with some resistance
    4. fully cooperative
  - Interest in the tasks:
    1. totally uninterested and displeased
    2. performs the tasks, but recklessly
    3. vinterested only in some tasks
    4. fully interested
  - Comprehension of instructions:
    1. does not understand the instructions
    2. understands the instructions, but needs many repetitions
    3. understands almost all instructions and requests assistance when necessary
    4. understands all instructions
  - Judgment and self-criticism regarding the procedure:
    1. claims to be unable and refuses to perform
    2. performs part of the tasks, claiming to be unable and displeased
    3. performs all tasks disregardfully
    4. performs all tasks without signs of self-criticism
  - Behavioral changes during the procedure:
    1. behavioral changes preclude the accomplishment of the tasks
    2. tasks are interrupted due to behavioral changes
    3. performs all tasks without interruption, but with anxiety and behavioral instability
    4. absence of behavioral changes
      Time:
      ( ) one-hour session ( ) two or more sessions
      Additional comments:
